# School counselors’ perceptions of virtual counseling in Lebanon: A qualitative study

**DOI:** 10.3389/fpsyg.2022.1083644

**Published:** 2023-01-12

**Authors:** Fatima Jaber, Anies Al-Hroub

**Affiliations:** Department of Education, American University of Beirut, Beirut, Lebanon

**Keywords:** virtual counseling, perceptions, technology, COVID-19, Lebanon

## Abstract

Virtual counseling has become an increased necessity as a result of the current pandemic as well as the need for methods that comply with the continuous changes and advancements. Studies conducted have shown that the perceptions of counselors of the benefits and barriers reflect whether or not the counseling service will be successful. This study is a descriptive research design following a qualitative approach. The study aimed to explore (a) the school counselor’s perceptions of the benefits and barriers they encounter when using virtual counseling, (b) the perceived differences between conducting virtual counseling and in-person counseling, and (c) whether participation in virtual counseling differs as a result of students’ characteristics in the perception of school counselors. Five private schools in the Beirut region were contacted as they were known to have virtual counseling programs. Both the schools and counselors were purposively chosen. Seven in-depth semi-structured interviews and one focus group discussion (FGD) were carried out with the study participants. Interpretational analysis was carried out to assess and analyze the obtained data. The results showed that through these experiences, counselors were able to develop new methods with regard to technology and other practices to limit the effects of obstacles faced. Counselors’ communication tools were also fine-tuned since they had to rely on unorthodox methods to understand students and deal with them better. Results showed that the benefits also have to do with time and method flexibility, the main barriers were issues of privacy, change in workplace dynamics, and lack of usage of tools. The findings were discussed in relation to four main themes: (a) service delivery adaptation, (b) working with technology, (c) counselor’s practices in delivering virtual counseling, and (d) preparation and training for virtual counseling. Being that it is qualitative research, the interpretational analysis was carried out to assess the obtained data. The results showed that the main benefits have to do with time and method flexibility, and the main barriers were issues of privacy and change in workplace dynamics. The results showed that the new changes and the counselor’s experience, along with the personal attributes of counselors and students, all influenced the efficiency of the counseling sessions.

## Introduction

While counseling has been established as a core aspect of school programs globally, this has not been the same case in Lebanon. It is essential to identify the achievements in counseling corresponding to a timeline to identify domain gaps. The counseling area’s advancements were slow to develop, and the developments that took place were mainly observed in career guidance (Ayyash-Abdo et al., 2010). There were early mentions and projects coordinated including the Career Guidance Center at the American University of Beirut in 1985 and that of the Hariri Foundation; however, they neither yielded as many results nor were the efforts reflected in school programs (Ayyash-Abdo et al., 2010). Virtual counseling has become an increased necessity as a result of the current pandemic and the need for methods that comply with continuous changes and advancements. Studies conducted have shown that the perceptions of counselors of the benefits and barriers reflect whether or not the counseling service will be successful. Virtual counseling is defined as counseling where a qualified counselor uses tools of technology to provide behavioral and mental health services to counselees in different locations. The integration of the Internet and counseling is a new occurrence known as virtual counseling. It has been given different labels, such as “cyber-therapy,” “e-therapy,” “virtual coach therapy,” and “cyber-counseling” to present virtual counseling (Smith and Reynolds, 2002).

There is slight information on using virtual counseling in the school setting. Counseling programs should take into consideration how frequently students feel like contacting a counselor online, or whether or not they would prefer face-to-face counseling. Youth might experience different challenges; however, they might not seek help for different reasons (Glasheen et al., 2013). Such information would be necessary for the counseling community to be able to serve the students in the school.

Various milestones, as well as events in history, have ignited the need for virtual methods, one of which is the outbreak of COVID-19. The onset of COVID-19 had major effects on various aspects of people’s lives, especially where students are concerned. Educational systems all around the world had to adapt in very little time to new means of education to ensure that students are provided with the necessary education (Aucejo et al., 2020). There was a wave of alternative options for every activity that required physical presence; accordingly, it is only fit that aspects related to students’ wellbeing, especially in such critical times, were developed.

Maples and Han (2008) stated that virtual counseling includes synchronous (video chatting and calling) and asynchronous (emails and texting) tools to address an individual’s needs. Each method of communication is used in a certain way. Steele et al. (2015) suggested that virtual counseling interactions can also be used with parents to address the personal/social, academic, and career domains.

Prior to the COVID-19 pandemic, the research identified several significant obstacles to therapeutic relationships brought on by the digital space and social media, such as therapist privacy concerns (clients frequently search for therapist/counselor personal information online, which inherently affects the therapeutic dynamic), virtual impingements (through which online discoveries alter the physical therapeutic relationship), and a desire to internalize digital versions of the counseling sessions. These obstacles were the same obtained during COVID-19 when reassessment was reconducted (Kotera et al., 2021). School counselors may have found themselves lacking support, training, and experience. The need for additional support during the pandemic, such as curriculum adaptation strategies, virtual lesson planning tools, addressing the loss of curriculum adaptation strategies, hands-on student learning, social and emotional support techniques, student engagement techniques in a virtual environment, and technical support, was difficult to pinpoint. To add on that, a recent study performed in New York on school counselors’ experiences in using virtual counseling reported results including the lack of access to students resulted in feelings of frustration, while another study was based on forced adjustment, navigating new technology, and guiding ethical concerns. The final study was about the challenge of addressing mental health complexities (Worth, 2022).

Research indicated the benefits and barriers to visual counseling from school counselors’ perspectives. Studies in Scotland and Finland, for example, explored counselors’ perceptions of the benefits of virtual counseling (Paterson et al., 2017). Paterson et al. (2017) stated that virtual counseling made counseling flexible, providing independent time and space to meet students’ perceived needs. Virtual counseling has also allowed students to anonymously enter discussions which enables disclosure for students who are timid about their personal matters. School counselors believe that virtual counseling also strengthened the bonding and counseling relationship (Paterson et al., 2017). Research in Kenya also showed that high-quality counseling services can stretch to areas that have insufficient resources to obtain trained counselors. Due to the financial status of families and their geographical locations, virtual counseling services could provide services for Kenyan students and take advantage of these school contexts and situate the tools that enable access to virtual school counseling (Pattison et al., 2012).

Paterson et al. (2017) explored other aims that researchers mentioned and that was to examine perspectives on virtual counseling held by CPD student counselors. Furthermore, they were asked to bring their strengths to the fore in this region. Four different orientations for virtual counseling arose from their responses, namely, technical organizational-centered, counseling-ethical, and developmental orientations. The guidelines offer a valuable viewpoint to look at ways in which student counselors view and construct their inner conceptions of virtual therapy. This provided a helpful framework for the discussion and creation of virtual counseling activities and the preparation and design of virtual counseling training. In addition, the joint relationship between Scotland and Finland has stated that the study’s results are not inherently country-specific. This yet again shows how such views can be similar or compatible in other countries. Lambie and Williamson (2004) found that the focus of counseling services was on character development, socially appropriate behaviors, and vocational planning. These aspects have shifted over time, moving from vocational and educational decision-making to personal growth, then to responsive services “at-risk” populations and ultimately focusing on developmental programs available for all students.

## Conceptual framework and research significance

The study’s objective was to contribute to both theory and practice, assisting in filling a gap in research that is rooted in the lack of in-depth studies carried out within the domain of virtual counseling. The concepts of this study were guided and illustrated by The American School Counseling Association’s (ASCA) model, which is an inclusive framework set for carrying out and evaluating school counseling programs. Studying counselors’ perceptions of virtual counseling can help provide reasons as to why there are only a handful of counselors who offer virtual counseling (Hoffman et al., 2000). Mallen et al. (2005) found that virtual counseling allows counselors to access populations in rural areas across the country; hence, such findings can provide further information for the preparation and training necessary for effective services to be provided to students.

As per new canvassing of experts’ views on what would be an expected and necessary change in the coming years is that people’s relationship with technology will deepen when more significant parts of the population will have to rely on digital connections for work, education, healthcare, and essential social interactions. Accordingly, it is vital to fill these gaps to access all necessary populations while being prepared with the tools needed (Atske, 2021).

The study also provided the training and coursework participating that virtual counselors underwent, which also acted as a guide for counselors new to the domain of virtual counseling (Lee, 2000). Open-ended questions were carried out with a number of counselors, to provide an in-depth perspective on the right approaches to providing virtual counseling. Observing and sharing counselors’ perspectives on barriers related to virtual counseling can also assist educational institutes in preparing for virtual counselor training. Learning through data collection tools about the most appropriate virtual technologies can offer facilitated and ongoing communication between school counselors and students of different cycles (Steele et al., 2015).

Successful virtual counseling will allow counselors to provide students of all cycles (K-12) with individualized data and direct feedback tailored to the student’s interests and values (Savitz-Romer et al., 2020). Conclusively, this study will be valuable and effective for the practice and preparation of future virtual counselors as well as the procedures used by different counseling organizations regarding ethics and legislation. Schools can use the study findings to provide supplementary information concerning the preparation of the virtual counselor’s skill training to ensure the effectiveness of the counseling service in school.

A study done in Georgia was also conducted by Hunter (2021) to add virtual school counseling knowledge and literature throughout the pandemic because unfortunately, there was restricted qualitative literature focusing on virtual school counseling. The ASCA has done much research to help conceptualize school counseling throughout all grade levels (K-12); however, in times of COVID-19, it was not initially validated. In October 2020, the ASCA surveyed 7,000 school counselors to collect data on daily challenges and the influence of the pandemic (Akgul et al., 2021).

More studies investigated the impact of COVID-19 with a concentration on teacher training and teachers’ experiences, however, the experiences of school counselors related to COVID-19 have yet to be explored (Savitz-Romer, 2021). According to the ASCA, school counselors are responsible for the social/emotional, career, and academic needs of students; therefore, the ASCA is making research pertaining to virtual school counseling and the COVID-19 pandemic vital for K-12 student achievement (Pincus et al., 2020).

The American School Counseling Association’s model includes programs that are equitable, data-driven, and respond to a diverse student population. The model includes four components that are the foundation, management, delivery system, and accountability to define how counselors and schools, whether on their own or collaboratively, support and work with students and their families (Bowers and Hatch, 2005).

According to Goodrich et al. (2020), the ASCA program has served as a reliable tool for school counseling nationwide and has recently delved into virtual counseling, providing guidelines for working in a virtual counseling environment. The ASCA progrm shed light on various aspects that were highlighted as barriers in the studies earlier. The program focuses on how counselors should simplify the methods of communication in order to avoid any misunderstandings that can take place in virtual counseling sessions. Students should also be proficient in using these tools to further facilitate the counseling program. The study also focuses on how the program should constantly be updated to provide the most efficient counseling. The most important point that was reiterated throughout the program is a collaboration with different departments as well as administration to provide comprehensive counseling to both students and parents.

The American School Counseling Association National model focuses on the usage of tools and highlights several benefits of virtual school counseling. Virtual school counseling has a great impact on school-aged students and adolescents. With the increased use of technology, school counselors can play a vital role in increasing awareness of the benefits and problems of using these tools of communication. These tools can affect the wellbeing of students because they can be a source of social support. School counselors who are confident in using the tools might also offer small or group technology interventions, model appropriate technology use, and address ethical considerations. The quadrant of the delivery system, which is an element of the ASCA model, would benefit from the increased use of technology to facilitate coordination, engagement, and communication. Virtual counseling can allow school counselors to provide individual as well as group counseling services by surpassing the time or space limitations found in traditional counseling. The use of asynchronous tools can allow school counselors to respond to students’ concerns throughout the day and allow them to have their regular sessions for academic and career-related purposes.

Despite the benefits of virtual school counseling, there were several drawbacks to it. A barrier to successful technology integration or usage is the spectrum of experiences and comfort levels using these tools that might exist between different generations. Virtual counselors should understand the problems or challenges resulting from virtual counseling, such as virtual aggression, concerns for privacy and safety, cyberbullying, as well as harassment. Internet anonymity and the lack of reduced auditory and visual cues can make it harder for students to feel comfortable communicating with the counselor during face-to-face sessions (Zamani et al., 2010).

Such measures apart from the organization being renowned in the domain of school counseling serve as enough reasons why this model should be followed to provide quality counseling. The ASCA National model can be used in the Lebanese context because the elements that make up the model have relevance wherever the school or school counselors operate. With the worldwide reach of digital technology, the applications to technology usage of this model can be used among school counselors globally.

## Research design

This study is a descriptive research design where qualitative approaches were utilized to explore and identify the perceptions of school counselors regarding the barriers they encounter when using virtual counseling. The qualitative research design is essential in educational research because it enables a deeper understanding of experiences, phenomena, and context. It allows researchers to understand human experiences through questions that cannot be easily put into numbers (Al-Hroub, 2011, 2014). Qualitative research gives comprehensive narratives of the circumstances that lead to participants’ focus on their perspectives toward an issue. This shows how qualitative research can show layers and provide a comprehensive understanding numbers cannot encompass (Al-Hroub, 2015). The researcher conducted semi-structured interviews including open-ended questions with private school counselors to provide insight into the obstacles counselors need to overcome to provide effective counseling sessions. A focus group discussion (FGD) was also designed to explore participants’ perceptions of virtual counseling. The study was conducted across different regions in Beirut with school counselors from different backgrounds to limit bias as much as possible and ensure validity.

### Research aims and questions

The aim of the study is 3-fold, i.e., (a) to explore the school counselor’s perceptions of the benefits and barriers they encounter when using virtual counseling, (b) to explore the perceived differences between conducting virtual counseling and in-person counseling, and (c) to determine if students’ characteristics differ in their participation in virtual counseling from the school counselors’ perspective. Three questions guided this research from the school counselors’ perspectives as follows: (a) what are the most contributing benefits and barriers that school counselors encounter during virtual counseling in private schools in the Beirut region?; (b) what are the perceived differences between conducting virtual counseling and in-person counseling?; and (c) does participation in virtual counseling differ as a result of students’ characteristics in the perception of school counselors?

### Study site and participants

The selected five private schools are located in the Beirut region. The schools have a virtual counseling program with English as their main language of instruction. A total of 11 counselors were selected using purposive sampling across all school levels (elementary, middle, and high school). Seven counselors took part in the semi-structured interviews, and six counselors participated in the FGD. The majority of the schools in Lebanon have only female counselors, and the sample of this study was limited to the number of counselors who had practiced virtual counseling. All study participants were women and had a similar level of educational background in earning master’s degrees in Educational Psychology. The counselors’ years of experience ranged from 1 to 15 years and their ages ranged from 27 to 48 years. Tables 1, 2 display the detailed demographic information of the study sample.

**Table 1:** Semi-structured interview participants’ demographic information.

School number	Participant assigned numbers	Counseling experience	Grade level	Education level	Age	Gender
5	1	1 year	Cycles 1&2	Masters	28	Female
5	2	5 years	Cycle 2&3	Masters	32	Female
3	3	3 years	KG &Cycle 1	Masters	27	Female
2	4	4 years	Cycle 2&3	Masters	30	Female
4	5	3 years	Cycle 1	Masters	29	Female
3	6	12 years	Cycle 2	Masters	42	Female
1	7	2 years	All cycles	Masters	34	Female

**Table 2:** Demographic information of school participants in focus group discussion.

School Number	Participant Assigned Numbers	Counseling Experience	Grade Level	Education Level	Age	Gender
1	A.S	2 years	All cycles	Masters	34	Female
4	B.R	3 years	Cycle 1	Masters	29	Female
3	C.F	4 years	KG & Cycle 1	Masters	30	Female
2	D.R	2 years	Cycle 2&3	Masters	34	Female
4	E.N	15 years	Cycle 1	Masters	48	Female
5	F.N	3 years	Cycles 1 & 2	Masters	28	Female

The participant’s privacy was protected by giving each one of them a code. The codes for the participants participating in the semi-structured interview were named counselor 1, counselor 2, … whereas the participants being part of the FGD started from the beginning of the alphabetical order with the first letter of their name.

Both the schools and counselors were purposively chosen. Purposeful sampling is a technique used largely in qualitative research for recognizing and collecting the most effective information with the utilization of limited resources. The process includes identifying and choosing individuals or groups of participants that are specifically well-informed on the phenomenon of interest (Palinkas et al., 2013). The counselors were chosen based on their performance in virtual counseling sessions and the schools were chosen based on their participation in having a virtual counseling program, and these choices are reasons yet again why purposive criterion sampling is carried out.

Ethical approval was obtained from the Institutional Review Board (IRB) at the American University of Beirut. Informed consent was obtained from the school principals and counselors. The researchers gave a full explanation of the study and assured the anonymity and confidentiality of all data collected.

### Data collection methods

The researchers conducted semi-structured interviews as well as an FGD to create an abundant description of the counselors’ perceptions of the barriers and benefits of virtual counseling. The participants were asked for permission to have their interviews and FGDs recorded and transcribed verbatim. They were offered to member check both their transcribed and recorded interviews to ensure internal validity and accuracy. After conducting semi-structured interviews and the FGD, the transcripts were analyzed and the themes were derived accordingly. These themes as will be mentioned below were the root of the research and helped identify key obstacles and challenges voiced by the counselors.

#### Semi-structured interviews

The semi-structured interviews were conducted with seven counselors from five schools and different cycles. The interview contains 10 questions that target the counselor’s experience, their use of technology, and their perception of virtual counseling. Probing questions were added to both sets of questions to attain the objectives of the study, which focus on the barriers perceived by the counselor for themselves and on behalf of the students. The interviews were conducted at the counselor’s convenience *via* Zoom, and each interview lasted for approximately 1 h.

#### Focus group interview

The focus group discussion included six counselors and lasted for 120 min through Zoom due to the restrictions during the COVID pandemic. The group represented different counselors from different schools (refer to Table 2), who discussed their perceptions and experiences on the barriers and benefits of virtual counseling.

### Data analysis

The data collected were analyzed using interpretational analysis. The recorded interviews and FGDs were transcribed verbatim by the researchers. The transcripts were coded using an open-coding method to identify the common themes that appeared in those transcriptions. The data were compared using the constant comparative method, where the similarities and differences of the data were derived. Later, the codes were organized into categories under general themes.

## Results

Virtual counseling was explained in different ways. There were different themes initiated after the semi-structured interviews and the FGD based on the interviewee’s responses. The study findings present four themes for the virtual counseling experiences: (a) service delivery adaptation, (b) working with technology, (c) counselor’s practices in delivering virtual counseling, and (d) preparation and training for virtual counseling.

### Service delivery adaptation during COVID 19

In all interviews, school adaptability in virtual counseling appeared as a major theme. Two main instigators led this overall needed change in the first place. There was discontinuity concerning school attendance because the first wave of COVID-19 that followed set a more fixed distance learning. The time needed for the counseling program to resume and in what form it did rely highly on the school’s perception of the need for counseling. The COVID-19 outbreak negatively affected the mental health and emotional, social, educational, and psychological wellbeing of young people (Karaman et al., 2021). Karaman et al. (2021) discussed that it was normal for the outbreak to cause anxiety and fear, but the anxiety and fear caused by the outbreak made it hard for students to fight the results of the outbreak. Therefore, the importance for school counselors to identify and respond to factors associated with anxiety helped reduce the students’ anxiety levels and contribute to fighting the pandemic.

Different counselors shared similar perceptions about the need for virtual counseling during the time of the pandemic. Counselor 7, for example, who was responsible for all cycles shared, “For students to be able to adapt to the changes of the environment, they should be able to voice their feelings and thoughts.” Therefore, “Due to the pandemic, there was a need for immediate counseling,” this sentiment represented all of the schools’ perceptions; however, the frequency of counseling sessions differed among schools and their priorities. One of the counselors summed up the mentioned in their statement: “This move was gradual, of course, however when corona struck it was then we started coming up with our own model for virtual counseling based on what professionals were doing around the world” (Counselor 3, KG & Elementary).

Counselor 2 (middle and high school) mentioned “It’s crucial to remember that there is a significant difference between in-person counseling and internet counseling. In order to create curriculums tailored specifically for virtual counseling, counselor educators require a programmer who is knowledgeable in virtual counseling.” Therefore, counselors had to find ways to start this program with strategies they were using in physical counseling.” Counselor 2 started by trying to incorporate one session per week that focused on coping strategies and mental health to help students cope with the new changes. Counselor 2 added, “We discussed topics that would help them cope with their daily lives.” The counselor also shared how the counseling sessions were tailored to fit what the students needed in the face of unexpected changes.

#### Change of school dynamics

As previously mentioned, some changes occurred in terms of content provided to better suit the occurring issues. Regarding the counselor’s schedules, there were clear shifts in the time they were expected to be available. Counselor 3 (KG and elementary level) shared: *“I was able to reach a bigger number of students at the same time, and I was able to be in two places at once, and students were happy* about it.” A.S (all cycles) agreed with Counselor 3 (KG and elementary level) and stated, “If properly structured, virtual counseling can be a huge outreach to people without the means due to physical problems, living in rural areas, or schedule complications.” The mentioned counselors had a positive experience; however, this was not the case for all. Carrying one’s job around even at home becomes harder for counselors to carry out their tasks effectively, being that they cannot divide their work and responsibilities at home. An elementary counselor shared struggles that she faced in trying to cater to both her house and school demands at the same time, being that there was no longer a partition between the two in terms of space and time. Counselor 6 stated, for example, “I have family issues I have to attend to while I’m at home, which I would not have to do if I were at school […] I have my children working at home.” These personal stressors were discussed by Savitz-Romer et al. (2020). Like all educators, school counselors tried to balance some personal stressors like their work and family demands, managing their mental health, and adjusting to new forms of technology.

In a study conducted by Worth (2022), a school counselor explained that it was even easier for students to access the counselor during school time, and helping students access technology resulted in time away from providing virtual counseling services to students. In the FGD, B.R. an elementary counselor shared, “With the change of schedules, counselors were expected to be there for students and their parents at any time even if that meant at 10 p.m.” Counselor 5 (elementary level) added that “Students come to my office to have their lunch, play board games, and have quick talks, when I’m on recess duty they come to me and talk to me.” While counseling in school was more structured and limited to school hours, being in school, however, facilitated students’ ability to approach counselors physically, even for a brief interaction. This facilitation ensures that the students do not hold in their emotions for too long and that even perceived trivial issues are shared with the counselor.

According to Courduff (2022), counselors felt that their schedules were full, and practices to take care of their mental health were a priority to best serve their students. There was an agreement among all 11 counselors concerning work changes and “overtime” work. During the FGD, two counselors C.F. and D.R. from different cycles (KG, elementary, and middle school levels) also shared that “The extension of work hours did not have to cater only to the needs of students.” They emphasized that their work was no longer constricted to school hours and the mortar space provided rather it became a full-time job.

### Lack of safe environment and space

Space was also an issue if they have their siblings in the same place or if they do not have a comfortable space to sit and be focused. Counselor 6 (middle school level) explained, “At home is different; there is the couch, the smell of the food from the kitchen, the doorbell ringing, and sounds from the street. The whole setting is not inducive for learning.” Students do not have a closed-mortar space for them to feel comfortable and free in their expression. The family being around students, as mentioned earlier, breaches the privacy they need. Counselor 4 who was in charge of middle school and high school elaborated that “Students felt invaded in their personal space because all of a sudden everyone can access them, at any time.” Despite the availability of interactive platforms, it is indisputable that the ability to communicate with students and the counselor in a traditional counseling setting cannot be matched in an online environment (Arrieta et al., 2021).

Chen et al. (2020) discussed that counselors and educators should set various guidelines to ensure a safe and respectful virtual environment. Some virtual counseling strategies include role-plays and mock counseling assignments that include information that is sensitive in nature; therefore, it is essential to promote confidentiality. During the FGD, some counselors discussed that they had to find alternative ways to connect with the students. C.F. (KG and elementary) and F.N. (elementary and middle school levels) shared alternative measures specifically for upper elementary and middle school, which included text messaging or even conducting sessions in the car to ensure privacy and comfort for students. Counselor 5 (elementary level) explained, “Some students want to see the counselor without their parents knowing; I cannot approach students without the consent of the parent.” Students in a synchronous environment may join the session in different settings, so it is vital to establish environmental guidelines that address the session’s safety and privacy (Chen et al., 2020).

### Preparation and training for virtual counseling

#### Experience with virtual counseling and technology

As can be seen here, the counselor’s familiarity and ease of using some of these platforms had to do not only with the schools they were counseling with but also with what they had experienced prior to this issue. Furthermore, although some of the interviewed counselors had a wide experience in counseling prior to this issue, they still shared some discomfort in switching to virtual methods. Counselor 6 (middle school) said, “This anxiety for the virtual counseling was meeting right after the first virtual meeting.” Personal choices and preferences influence a counselor’s overall experience. Counselor 3 (KG and elementary) shared that “while it is an important matter to use technology tools professionally, it wasn’t a tool that she liked or preferred personally.” Although a lot have stated obstacles and issues in the shift, the positives and necessity of virtual counseling were inevitable. Deslonde and Becerra (2018) stated that according to research, counselors are more likely to accept using new technology if they perceive it as easy to use. Moreover, perceived ease of technology use is determined when a counselor considers using such a system free of effort.

#### Practice enhancement and motivation

Working in a virtual counseling environment has improved all of the participants’ counseling abilities and practice. Moreover, recommendations for better practice are also examined so that counselors can have better preparation before practicing virtual counseling (Poh Li et al., 2013). A.S. (all cycles) started by stating, “School counselors should be trained the provide virtual counseling. To become proficient in using this modality, we should be prepared and competent. This is the aim of our field present and future.” Counselor 7 (all cycles) continued with sharing her own training and stated, “The following websites where she underwent different training and workshops aimed solely at virtual counseling with regards to student’s wellbeing.” All counselors shared a keen interest in learning to use different technology platforms and attending more training aimed at enhancing their virtual counseling skills. School counselor training differed from one school to the other; therefore, developing school counselor abilities in the use of information and communication technology (ICT) should be concerned with helping them build familiarity with ICT applications and awareness of the potential contribution it has to offer the virtual counseling services (Deslonde and Becerra, 2018).

### Counselor’s practices in delivering virtual counseling

#### Methods used by counselors during virtual counseling sessions

Strear et al. (2020) discussed that school counselors started adapting their techniques to meet the immediate needs of their school communities as they attempted to interact with students and their families. Many counselors started to update their strategies giving students and families access to messages, calls, and video appointments. Some virtual engaging tools were brain games, puzzles, exercise body games, and artistic activities were done virtually. Some counselors shared that the methods they used did not differ but only included more technology. Counselor 6 (middle school) stated “Sometimes I start by playing a virtual game like Kahoot (It is a game-based learning platform for teachers and students, it is used to generate multiple quizzes) just to break the ice. Sometimes it would be “guess who,” these games are available online.” This did not apply to other counselors. While most of them shared that they were trying to connect with the students the way they did in school, they shared alternative methods they utilized to engage and connect with students virtually. Counselor 1 who was responsible for elementary and middle school shared that *“I used both humor and shared personal experience to establish a comfortable environment.”* The same method was voiced by Counselor 5 (elementary level) who allowed students to focus on relational skills needed based on their age. These virtual tools, which focused on student’s academic, social, and emotional growth as well as their readiness for a variety of postsecondary options, are just a few examples of school counselors’ leadership, knowledge, and creativity, and they will make a significant and lasting contribution to the field (Strear et al., 2020).

#### Roles and responsibilities of counselors

The lack of guidance offered to counselors developed as a result of losing time in the move to an online support system and resorting to asking counselors to fill in logistical gaps for school administrators, such as attendance tracking and other tasks that fail to leverage counselors’ exclusive expertise in mental health or student support (Savitz-Romer et al., 2020). During the interviews with the counselors, it was seen that some of the core aspects that have influenced the obstacles addressed were the roles and responsibilities of counselors. It was shown through discourse analysis how obstacles varied as a result of the roles and responsibilities delegated to the counselors, and how they tried to adapt to such changes. Counselor 1 (elementary and middle school levels) stated, “As a counselor, my role is to support my students, socially, emotionally, psychologically, and academically.” These were among the tasks the counselors emphasized that they carried out. “Some counselors shared that it was overwhelming even though some tasks did not increase, but the role itself was more demanding.”

In a study conducted by Savitz-Romer et al. (2020), school counselors also reported a lack of direction and leadership from school and district leaders. The provision of tasks and the specificity of each had to do with the school’s administration and philosophy. Requirements of the counselors varied, while a few counselors shared that the requirement was already established; others like Counselor 6 (middle school level) shared that “it was hard to draw the line or establish on their own what criteria to follow since that was not set.” Counselor 5 (elementary level) indicated, “We are not teachers, and we are not in the administration. A lot of times, I find myself where I do not have much work, so I think the counselor has to support teachers’ parents and students.” The communication with parents and presentation to students about who the counselor was and their roles allowed counselors to abide by their tasks and set required fine lines. Some of the tasks counselors continued to do even in virtual counseling were meeting with parents and new applicants to establish a connection with them and, as mentioned, setting a fixed frame for the counselors’ roles.

Another continued activity was the orientation day which counselors would usually carry out, within the pandemic, counselors committed to conducting them, however, virtually. Counselor 4 (middle school and high school) stated, “Sometimes students require a referral to a psychiatrist outside school because sometimes a child might have learning needs such as ADHD, or dyslexia. I would coordinate with the therapist that accesses the students and coordinate with the teachers to help them adjust to the accommodations and modifications.” Counselors worked internally and externally to ensure that students’ needs were provided. Counselor 1 (elementary and middle school levels) added that “The drawbacks of virtual counseling are increased liability, some counselors were not able to respond to the specific crisis as in face to face counseling, and the incapability to handle a crisis.”

#### Types of technological platforms used in counseling

Having to conduct most sessions virtually required that the counselors find facilitating tools to enrich their sessions and collect data on their students. Counselor 1 (elementary and middle school levels) explained, “Some of these tools were used before, and updated versions were utilized, and in several cases, completely novel platforms and tools were used to facilitate virtual classes.” This supports a study conducted by Brewington and Kushner (2020) where various school counselors noticed that sending emails, designing multimedia presentations and webpages, developing newsletters, and retrieving information from schools’ student information systems are relative functions that help the counseling sessions.

In contrast, counselor E. N (elementary level) shared doubt about elementary students’ ability to reach out virtually *via* email or seesaw. According to a study conducted by Worth (2022), counselors explained how students would often keep their cameras off and not respond to different methods of communication such as emails, phone, and messages.

These opposing views had to do also with counselors’ experiences with the students and how they viewed these tools to be reflected in students’ behavior, performance, and responsiveness to counseling.

#### Limitations of a virtual counseling session

Currie (2010) explained that despite virtual counseling’s benefits to students’ wellbeing, there were various limitations. Different limitations discussed were students’ readiness, organization and administration of the program, facilitator’s ability to replace non-verbal cues with textual techniques, and technological training and support. Such limitations affected the attainment of virtual counseling sessions. There were various limitations discussed by counselors regarding their virtual counseling experiences with students. The issues outlined by counselors were many but had a similar theme, which is that virtual counseling cannot replace in-person counseling. Counselor 5 (elementary level) said, “Some students preferred typing so that the counselor would not know what they are feeling from the tone of their voices” Counselor E.N (elementary level) also added that “Some students had a great experience in writing out their feelings, it was the same as journaling but they had their counselor as a listener to tell them what they heard or read between the lines.” D.R who is responsible for middle school and high school shared tips on how she tried to make the student comfortable; she explained that “by providing them with the autonomy in choosing the way they are comfortable in communicating, a need that aligns with their age group.” With the struggles faced, counselors tried to come up with tactics to limit these obstacles; nonetheless, the consensus was that virtual counseling cannot replace in-person counseling.

Students who could not communicate privately with the counselor either omitted part of the information or had to use other methods for privacy matters which yet again made it difficult for the counselor to understand what the students were going through. Certainly, there is a lag in catching up with the students. Counselor 7, who is responsible for all cycles, emphasized, “When counselors see a student at school, they look for them and catch up with them, particularly in the hallway, however virtually such communication can be slowed down due to different factors.” Baker and Ray (2011), in contrast, highlighted a disadvantage that the tools used in virtual counseling might impede the process, which results in clarification problems rather than focusing on the counselee’s goals. Written communication can allow the counselee to revise their statements without worrying about them coming out wrong (Baker and Ray, 2011).

Finally, one of the most “specific” issues was related to the internet and electricity. Virtual live sessions were provided regularly to students as much as possible; however, many considered this approach to be a failure because of the weak Internet connection and the lack of electricity in the majority of the Lebanese regions (Anouti and Rouadin, 2020). Internet and electricity were a barrier; even several siblings having a Zoom meeting at the same time might break the connection. Counselor 1 (elementary and middle school levels) explained, *“The access to computers and devices at our school was also an issue.”* Until now, many students worldwide do not personally have a desktop computer or a laptop or even access to one. This issue is yet to be solved in many countries (Li and Lalani, 2020).

There are different views on privacy and confidentiality in virtual counseling. Counselor 6 (middle school level) indicated, *“For most, virtual counseling offers students privacy to say what they want without being seen by their friends or teachers.”* This, however, does not eliminate limitations that counselors shared they faced “one of the students would always delete the emails he would send to make sure that his parents do not read the emails.” Having virtual counseling prevented some students from being able to communicate with the counselors comfortably because even if they had a private space, parents still have access to their digital devices.

#### Students profile

The social constructions give space for women to be in touch with their feelings, which is reflected in how women are more likely to reach out for help than men. The themes discussed in higher school differed between the male students and the female students. The female students were more willing to discuss their mental health and challenges, but the male students were cautious about discussing their mental health. Even if they are hesitant to open up during counseling sessions, students contact counselors more in person because there is no need to send emails or schedule appointments.

A study carried out by Tsan and Day (2007) was conducted to focus solely on the role of gender in determining students’ perception of counseling. The study found a robust positive relationship between one being male and their likeliness to choose virtual counseling. This was connected with the issue of masculinity.

Some studies explain that students prefer virtual learning, particularly those who may feel shy and insecure, those with learning difficulties, those who find public speaking difficult, and those who are unwilling to talk in class (Nasution et al., 2021). To support this notion, some counselors explained that students’ approach to counseling relies more on personal traits and preferences rather than gender. B.R. (elementary level) mentioned, *“Students who are shy communicate and share more in virtual counseling, although some were anxious to open the camera, this yet again made the virtual counselor closer to an in-person session.”* It has always worked with learners with difficulties. The distance approach reduced some of the stress they felt when communicating in person with students. Interest in attending counseling sessions had to do with the schools’ approach to it.

Students who were grade-oriented found counseling sessions useless since they could not obtain grades from attending. Such issues limited students’ proactive participation in some schools. Students in cycle 2 were more likely to attend counseling sessions than those in cycle 3 due to several factors. To begin with, students in cycle 2 are more likely to be referred than those in cycle 3.

Students in cycle 3 also have a tougher attitude toward counselors since they are at an age where they are trying to fit in, and attending counseling still has a bit of a stigma to it in schools. Counselor 7 who was responsible for counseling all cycles explained, “The attitudes of students in Cycle 3 are tougher since over the year they are less likely to be accepting of “counseling” and its importance.”

Tsan and Day (2007) also discussed how physical development and self-perception also played an essential role in students’ perceptions of virtual counseling. During the FGD, counselors discussed an important matter concerning students who were maturing and becoming self-aware of their physical appearance. A discussion among the counselors A.S (all cycles) and E.N (elementary level) occurred where it was shared that “Hesitation to show one’s face was a result of them feeling watched or just being uneasy in that setting. The hormones and physical appearance of students of this age, and students start reflecting and judging themselves.” Students who are self-conscious about their appearance cannot hide their faces and, at some point, may forget how they appear or may be able to ignore what worries them about their appearance.

### Summary of findings

According to the counselors, the difference in virtual counseling challenged the initial environment by having them and students adapt to a novel experience, specifically concerning their work schedule and spaces they shared with a student that they no longer did. In addition, the counselors have stated benefits across different issues, while a lack of privacy was present with regards to this new “environment”; the counselor shared that they were able to develop new methods that are both related to technology and counseling practices. Furthermore, counselors shared that student characteristics have played a role in determining the student’s responsiveness to counseling. Students in the middle and secondary levels were more capable of obtaining privacy and access to different means of technology, in one way or another than those younger. The methods of counseling students preferred also relied on their personal preference, and the seeking for counseling was ultimately influenced by gender where female students were more likely to ask for sessions than male students.

In all of the interviews, environmental adaptability in virtual counseling appeared as a major subject. Within this issue, the time the counseling programs needed to resume after the initial lockdown relied on the school’s perception of the need for counseling. The programs were adapted in all the mentioned schools to fit the changes occurring. Such changes also influenced the counselor’s roles and availability. The counselors had more roles to carry out and, in most cases, were required to be available out of expected work hours. For certain counselors, their roles did change however the work hours were shortened; either way, the new changes influenced both the time and roles expected. Virtual counseling made it more difficult to communicate with the counselors due to a lack of privacy at home along with electricity and Internet connection issues, which impeded proper communication from taking place. Various solutions were created on both ends and over the years, counselors and students found new ways to facilitate communication. Counselors who had different years of experience with regard to technology facilitated the use of technology for some counselors over the ones with less experience. Nevertheless, they all had a positive experience with the tools they had to use. Counselors utilize different methods and counseling approaches according to the needs of both the students and parents. The counselor’s roles were not bound solely by what the school provided; counselors would extend their search beyond the school parameters to ensure that the student’s needs are provided. All counselors agreed that the virtual counseling experience improved their counseling abilities and practices. Counselors did agree that certain attributes influenced students’ readiness to reach out to counselors and engage with them; the attributes included age and gender. Such issues all played a role in the results obtained.

## Discussion

The research emphasized how virtual counseling creates a more flexible space for both counselors and students. This was further seen by what counselors shared. Some counselors shared that they were more flexible with having the counseling sessions done whenever they could, even after school schedule. The contact with the counselor is conducted “one on one” which ultimately makes the students feel that they have the counselor’s full attention.

It was worth considering not only the counselors’ experience but also its role in their counseling education. Counselors were not exposed to the various platforms that were suggested by schools. Such issues, however, were taken as challenges by the counselors and not threats. Counselors discussed the development of new skills and the refinement of existing ones.

The importance of technology integration as had been experienced first-hand by the counselors is supported by Paterson et al. (2017) who stated that there should be an increased focus on the integration of technology with regards to counselors’ roles because ultimately adapting well to these tools facilitates and improves their function as counselors.

Counselors’ understanding regarding what tools students are most comfortable with and choosing methods that would facilitate communication would not only encourage students to approach counselors but also push them to come back a second time. This also aligns with views of King et al. (2006) where they shared that choosing effective tools will let the session more efficient and engaging. Furthermore, understanding the factors that would encourage and hinder students’ motivation to use the Internet can increase access to resources for this vulnerable group.

Counselors’ perspectives on the benefits of virtual counseling have been highlighted in studies conducted in Scotland and Finland. Virtual counseling made counseling more flexible, allowing students to fulfill their own needs in their own time and space. Virtual counseling has also allowed students to participate in talks anonymously, allowing those who are shy about sharing personal information to do so. Virtual therapy, according to school counselors, increased the bonding and counseling relationship (Paterson et al., 2017). The aim of counseling is cross-cultural and they were also the same aims counselors wanted to adhere to in physical counseling and virtual counseling. One of these aims was the establishment of a bond between the counselor and the students.

In the “kids’ helpline” study, the counselee’s age played a significant effect on which tools they chose (King et al., 2006). Some counselors shared that students in the elementary division did not know how to reach the counselors virtually through the school’s suggested platforms. Generally, they had similar issues that the counselors had to work on. Counselors did agree that there were specific personal preferences among students; however, there were common issues that they could work on according to age groups. Not only was the topic of interest common according to the age group, but there were also similar technical issues that were experienced by specific age groups. As students grew older, they start becoming more self-conscious about their appearance.

There is a social construction that allowed women to be in touch or open about their feelings more than men. It was agreed by different counselors that men were not given the accepting space; therefore, when provided by the counselor, they attended and were committed to it. Male students were more referred by teachers or counselors in the elementary division, whereas female students tended to approach the counselor proactively more. In the high school divisions, female students were more likely to express and talk about their mental health challenges, but male students were more cautious in discussing them. With the external pressures that society throws on men when it comes to discussing their emotions, they will certainly find it easier to do so in an environment that affords a sense of anonymity. Karaman et al. (2021) conducted a study that focused on students’ psychological symptoms and explained that there was a difference between male students and female students. Results indicated that female students in high school have higher scores on anxiety, depression, and negative self-concept. In other words, female students experienced virtual counseling more than male students.

Counselors were actively aware and engaged in ethical matters. Ethical issues about confidentiality were among the most reported issues. Counselors shared that they tried to find different methods to ensure confidentiality and privacy, especially with students facing issues with their parents and having no “private” means of communication. These notions were supported by Kit et al. (2017) when stating that counselors should address ethical concerns about virtual counseling privacy by informing students of confidentiality restrictions, technical limitations, and methods for maintaining the confidentiality and providing emergency support.

Counselors shared that they used both synchronous and asynchronous methods to cater to students’ needs. Virtual counseling was not a single method but rather a combination of the mentioned tools to ensure that efficient and suitable counseling takes place. Virtual counseling, according to Maples and Han (2008), ultimately comprises both synchronous (video chatting and calling) and asynchronous (emails and texting) techniques for addressing an individual’s needs.

Career guidance is vital for middle and high school children, and therapeutic software can be utilized to address their concerns. The counselors for the mentioned cycles tried to focus on “career guidance” as well as discussions of social issues that aligned with the issues students at this age face.

Counselors addressed that several issues faced by students had to do with the parents. Accordingly, sessions with parents were also viewed as crucial in carrying out counseling. Such a call to action is also backed up by Steele et al. (2015), who shared that virtual counseling exchanges with parents are possible. Regularly, the counselor would engage with parents and their children to address personal/social, academic, and career areas. It was further stressed by Khansa (2015) who claims that it would be beneficial for counselors to engage parents and teachers in workshops and seminars.

Counselors who had no previous virtual counseling experience shared that in the first virtual sessions, they felt as if it is the first time, they were counseling albeit them having several years of experience. This initial discomfort influenced their ability to carry out their work efficiently. Counselors also added that becoming more confident in their use of virtual tools assisted them in providing better counseling and allowed them to be more attentive to the student’s needs. Steele et al. (2015) stressed that by sharing that counselors should be comfortable and confident in their use of virtual communication tools.

According to research, primarily obtained knowledge, personal opinions, Internet accessibility, secrecy, and privacy all had positive and negative impacts. Internet accessibility was one of the issues that may have influenced “virtual counseling to be a negative experience” (Ayyash-Abdo et al., 2010). Counselors were ultimately all able to agree that this was a positive experience because they were able to take the negative and turn it into an opportunity to learn more, hence a positive experience.

Students who proactively asked for sessions tried different methods to ensure that a session with the counselor would take place. Even concerning privacy, different students would find different methods to contact the counselors; one of the examples provided by the counselor is how students would have the session in the car in order to have a video call with the counselor to follow up on the issue. Distressed students were more concerned about the counseling’s effectiveness than the skills and instruments required to conduct a successful virtual counseling session. The sentiment that was expressed by researchers was as mentioned seen to a certain extent with students (Li and Leung, 2020).

According to counselors, students were more likely to seek counseling services that were easy to obtain, especially in teenage groups where students were less likely willing to approach counselors for help (Kotera et al., 2021). Counselors seek to provide “easily accessible services” by yet again providing asynchronous ad synchronous methods. This yet again is supported by research obtained, where a lot of focus was on how “online” counseling may free counselors and students from time, location, and space constraints. Such benefits also influenced students’ decisions to seek counseling, as seen by the students’ decreased threshold for seeking help.

### Limitations, implications, and conclusion

There were some limitations in this study. First, the sample size was small and represented only female counselors. Second, the data were collected through virtual interviews, with few opportunities to see school counselors practice in the field. Finally, the selected counselors represented private schools only in the area of Beirut, the capital of Lebanon.

The shift to remote schooling left school counselors very much in the dark about their role and responsibilities and it restricted their capabilities to deliver school counseling services in some schools. The interviews conducted with school counselors showed that they did their absolute best to uphold their relationships with the students and continue to find ways to carry out their duties, getting creative in the process. There are specific recommendations for support and district leaders who are willing to sustain their commitment to students’ wellbeing and postsecondary readiness when schools shift remotely. It is essential to establish a clear plan for virtual counseling services and support, and communicate it widely, especially to families and other members of the school community. School counselors’ input can help identify counseling-related policies and practices that are translated to virtual or hybrid contexts. From the conducted interviews, counselors found ways to integrate their services into virtual. Structured time should be created for counselors to meet with students and even their families. School leaders should call on all school members and staff to track down students and interfere and monitor students’ attendance, which will leave school counselors to use their time to check in with students, deliver resources for managing their wellbeing, and conduct other counseling-specific activities. Solutions should be pursued to enhance virtual counseling while considering confidentiality and privacy issues. It should be ensured that counselors have access to resources, training, and support. It is also important to inquire about what kind of assistance counselors need to interact with students and deliver counseling *via* virtual platforms. Furthermore, counselors should be provided with training throughout the academic year, especially in times of rapid change, and make sure counselors are given time to recharge.

One aspect of the virtual school counselor’s challenge is that there was not a lot of knowledge and information on virtual counseling in counselor education. Much of the research done on this topic concentrated on ethical issues and whether schools should implement them or not. There was less practical assistance for the school counselors who had no choice but to practice in a virtual setting, especially for the first-year counselors. Counselors had minimal outside resources to turn to for advice on how to practice or improve their skills. Universities should acknowledge their existence and give the field of virtual school counseling the same thoughtful, critical research as it does to all the other relevant subjects. Counselor educators in the field of virtual counseling should acknowledge the growing impact communications technology has when it comes to supporting and communicating with one another.

In conclusion, several aspects were discussed, and shared ideas were obtained. There should be a greater emphasis on technology integration in counselor jobs because, in the end, being able to adapt well to these tools helps and improves their function as counselors. Such changes would rely on students’ ages since such criteria dictate what tools and methods work. Virtual counseling uses both synchronous (video conferencing and calling) and asynchronous (emails and texting) strategies to meet a person’s requirements, flexibility with regard to meeting time, and even the method serves as the primary method. Confidentiality and privacy are the significant barriers and challenges counselors would face; however, having no alternative option pushed counselors to come up with solutions to deal with such issues. Counselors should be comfortable and confident in their use of virtual communication platforms and should provide the same comfort for students for such sessions to be efficient. It was found to be strongly associated with better levels of comfort with the proposed service. The age group played a significant role in identifying the type of counseling students would respond to. Mainly, middle schoolers responded best to the tackling of social issues as a group since these were the issues they would be dealing with. Gender was also an essential factor since it was asserted by counselors that women were more likely to approach counselors than men because of gender norms and social constructions attached to them. Ultimately, creating facilitated means for reaching out to counselors, be it in technology or even addressing issues students would identify with, would push students to approach counselors, commit to the session, and ultimately assist in the counseling being more efficient.

## Data availability statement

The raw data supporting the conclusions of this article will be made available by the authors, without undue reservation.

## Ethics statement

The studies involving human participants were reviewed and approved by The American University of Beirut. The patients/participants provided their written informed consent to participate in this study.

## Author contributions

All authors listed have made a substantial, direct, and intellectual contribution to the work and approved it for publication.

## Conflict of interest

The authors declare that the research was conducted in the absence of any commercial or financial relationships that could be construed as a potential conflict of interest.

## Publisher’s note

All claims expressed in this article are solely those of the authors and do not necessarily represent those of their affiliated organizations, or those of the publisher, the editors and the reviewers. Any product that may be evaluated in this article, or claim that may be made by its manufacturer, is not guaranteed or endorsed by the publisher.

## References

[ref1] Akgul T. Brown J. Karch L. (2021). The personal and professional impact of covid-19 on school counselors: an exploratory study. Interact. J. Glob. Leadersh. Learn. 2, 1–31. doi: 10.55354/2692-3394.1024

[ref2] Al-Hroub A. (2011). School dropouts in Palestinian refugee camps in Lebanon. Issam Fares Institute for Public Policy and International Affairs (IFI) & United Nations Relief and Works Agency for Palestine Refugees (UNRWA). Available at: https://www.aub.edu.lb/ifi/public_policy/pal_camps/Documents/research_reports/20111212ifi_pc_unrwa_research_report01_hroub_english.pdf

[ref3] Al-Hroub A. (2014). Perspectives of school dropouts’ dilemma in Palestinian refugee camps in Lebanon: an ethnographic study. Int. J. Educ. Dev. 35, 53–66. doi: 10.1016/j.ijedudev.2013.04.004

[ref4] Al-Hroub A. (2015). Tracking dropout students in Palestinian refugee camps in Lebanon. Educ. Res. Q. 38, 52–79.

[ref5] Anouti M. Rouadin N. (2020). The virtual learning experiment in the intermediate and secondary schools in Lebanon during the coronavirus (COVID-19) crisis. Int. J. Adv. Res. Eng. Technol. 7, 14466–14485.

[ref6] Arrieta G. S. Valeria J. R. Belen V. R. (2021). Counseling challenges in the new normal: inputs for quality guidance and counseling program. Counsellia 11, 71–85. doi: 10.25273/counsellia.v11i1.8802

[ref001] Atske S. (2021). Experts say the ‘new normal’ in 2025 will be far more tech-driven, presenting more big challenges. Pew Research Center: Internet, Science & Tech.

[ref7] Aucejo E. M. French J. Ugalde Araya M. P. Zafar B. (2020). The impact of COVID-19 on student experiences and expectations: evidence from a survey. J. Public Econ. 191:104271. doi: 10.1016/j.jpubeco.2020.104271, PMID: 32873994 PMC7451187

[ref8] Ayyash-Abdo H. Alamuddin R. Mukallid S. (2010). School counseling in Lebanon: past, present, and future. J. Couns. Dev. 88, 13–17. doi: 10.1002/j.1556-6678.2010.tb00143.x

[ref9] Baker K. D. Ray M. (2011). Virtual counseling: the good, the bad, and the possibilities. Couns. Psychol. Q. 24, 341–346. doi: 10.1080/09515070.2011.632875

[ref002] Bowers J. Hatch T. (2005). The ASCA national model: A framework for school counseling programs. (2nd ed.). American School Counselor Association.

[ref10] Brewington M. Kushner J. (2020). School counselor perceptions of a comprehensive school counseling model and implications for practice. Administ. Issues J. Educ. Pract. Res. 10, 33–45. doi: 10.5929/2020.10.2.3

[ref11] Chen S.-Y. Wathen C. Speciale M. (2020). Online clinical training in the virtual remote environment: challenges, opportunities, and solutions. Profess. Counsel. 10, 78–91. doi: 10.15241/syc.10.1.78

[ref12] Courduff J. (2022). “Special educators' experiences pivoting from face-to-face to virtual during COVID-19: A phenomenological study.” in *Proceedings of the 2022 AERA Annual Meeting*.

[ref13] Currie N. S. (2010). Virtual counseling for students enrolled in online educational programs. Educ. Consider. 37, 22–26. doi: 10.4148/0146-9282.1153

[ref14] Deslonde V. Becerra M. (2018). The technology acceptance model (TAM): exploring school counselors’ acceptance and use of Naviance. Profess. Counsel. 8, 369–382. doi: 10.15241/vd.8.4.369

[ref15] Glasheen K. Campbell M. A. Shochet I. (2013). Opportunities and challenges: school guidance counselors’ perceptions of counseling students online. Aust. J. Guid. Couns. 23, 222–235. doi: 10.1017/jgc.2013.15

[ref003] Goodrich K. M. Kingsley K. V. Sands H. C. (2020). Digitally responsive school counseling across the ASCA national model. Int. J. Adv. Couns. 42, 147–158. doi: 10.1007/s10447-020-09396-9

[ref16] Hoffman D. L. Novak T. P. Schlosser A. (2000). The evolution of the digital divide: how gaps in internet access may impact electronic commerce. J. Comput.-Mediat. Commun. 5, 233–245. doi: 10.1111/j.1083-6101.2000.tb00341.x

[ref17] Hunter T. M. (2021). Virtual school counseling amid the COVID-19 pandemic. Doctoral Dissertation. Available at: https://digitalcommons.liberty.edu/cgi/viewcontent.cgi?article=4037&context=doctoral

[ref18] Karaman M. A. Eşici H. Tomar İ. H. Aliyev R. (2021). Covid-19: are school counseling services ready? students' psychological symptoms, school counselors' views, and solutions. Front. Psychol. 12:647740. doi: 10.3389/fpsyg.2021.647740, PMID: 33868121 PMC8044295

[ref19] Khansa R. (2015). Teachers’ perceptions toward school counselors in selected private schools in Lebanon. Procedia Soc. Behav. Sci. 185, 381–387. doi: 10.1016/j.sbspro.2015.03.411

[ref20] King R. Bambling M. Lloyd C. Gomurra R. Smith S. Reid W. (2006). Online counselling: the motives and experiences of young people who choose the internet instead of face to face or telephone counselling. Couns. Psychother. Res. 6, 169–174. doi: 10.1080/14733140600848179

[ref21] Kit P. L. Teo C. T. Tan M. Park Y. (2017). Singaporean counselors’ virtual counseling experiences with children: an exploratory qualitative study. J. Asia Pac. Counsel. 7, 141–168. doi: 10.18401/2017.7.2.3

[ref22] Kotera Y. Kaluzeviciute G. Lloyd C. Edwards A.-M. Ozaki A. (2021). Qualitative investigation into therapists’ experiences of online therapy: implications for working clients. Int. J. Environ. Res. Public Health 18:10295. doi: 10.3390/ijerph181910295, PMID: 34639594 PMC8507863

[ref23] Lambie G. W. Williamson L. L. (2004). The challenge to change from guidance counseling to professional school counseling: a historical proposition. Prof. Sch. Couns. 8, 124–131.

[ref004] Lee C. C. (2000). “Cybercounseling and empowerment: bridging the digital divide,” in Cybercounseling and Cyberlearning: Strategies and Resources for The Millennium. eds. Bloom J. W. Walz G. R. (Alexandria, VA: American Counseling Association), 85–93.

[ref24] Li C. Lalani F. (2020). The COVID-19 pandemic has changed education forever. This is how. The World Economic Forum. Available at: https://www.weforum.org/agenda/2020/04/coronavirus-education-global-covid19-online-digital-learning/

[ref25] Li T. Leung C. (2020). Exploring student mental health and intention to use virtual counseling in Hong Kong during the COVID-19 pandemic. Psychiatry Clin. Neurosci. 74, 564–565. doi: 10.1111/pcn.13117, PMID: 32686896 PMC7405056

[ref005] Mallen M. J. Vogel D. L. Rochlen A. B. (2005). The practical aspects of virtual counseling: ethics, training, technology, and competency. Couns. Psychol. 33, 776–818.

[ref26] Maples M. F. Han S. (2008). Cybercounseling in the United States and South Korea: implications for counseling college students of the millennial generation and the networked generation. J. Couns. Dev. 86, 178–183. doi: 10.1002/j.1556-6678.2008.tb00495.x

[ref27] Nasution A. K. Surbakti A. H. Zakaria R. Wahyuningsih S. K. Daulay L. A. (2021). Face to face learning vs blended learning vs online learning (student perception of learning). J. Phys. Conf. Ser. 1783:012112. doi: 10.1088/1742-6596/1783/1/012112

[ref28] Palinkas L. A. Horwitz S. M. Green C. A. Wisdom J. P. Duan N. Hoagwood K. (2013). Purposeful sampling for qualitative data collection and analysis in mixed method implementation research. Adm. Policy Ment. Health Ment. Health Serv. Res. 42, 533–544. doi: 10.1007/s10488-013-0528-y, PMID: 24193818 PMC4012002

[ref29] Paterson S. Laajala T. Lehtela P. (2017). Counselor students' conceptions of virtual counseling in Scotland and Finland. Available at: https://www.tandfonline.com/doi/abs/10.1080/03069885.2017.1383357

[ref30] Pattison S. Hanley T. Sefi A. (2012). “Virtual counseling for children and young people: using technology to address the millennium development goals in Kenya” in Virtual Guidance and Counseling: Toward Effectively Applying Technology. eds. Popoola B. Adebowale O. (Pennsylvania, PA: IGI Global), 135–151.

[ref31] Pincus R. Hannor-Walker T. S. Wright L. Justice J. (2020). Covid-19’s effect on students: how school counselors rise to the rescue. NASSP Bull. 104, 241–256. doi: 10.1177/0192636520975866

[ref32] Poh Li L. Jaladin R. A. M. Abdullah H. S. (2013). Understanding the two sides of online counseling and their ethical and legal ramifications. Procedia Soc. Behav. Sci. 103, 1243–1251. doi: 10.1016/j.sbspro.2013.10.453

[ref33] Savitz-Romer M. (2021). “When the kids are not alright: school counseling in the time of covid-19.” in *Proceedings of the 2021 AERA Annual Meeting*.

[ref34] Savitz-Romer M. Rowan-Kenyon H. T. Nicola T. P. Carroll S. Hecht L. (2020). Expanding support beyond the virtual classroom: Lessons and recommendations from school counselors during the COVID-19 crisis. Harvard Graduate School of Education & Boston College Lynch School of Education and Human Development.

[ref35] Smith S. D. Reynolds C. (2002). Cyber-psychotherapy. Ann. Am. Psychother. Assoc. 5, 20–23.

[ref36] Steele T. M. Jacokes D. E. Stone C. B. (2015). An examination of the role of virtual technology in school counseling. Prof. Sch. Couns. 18, 125–135. doi: 10.5330/prsc.18.1.428818712j5k8677

[ref37] Strear M. Duffy H. Sunde A. (2020). When schools go dark, school counselors shine: School counseling during a global pandemic. American Institutes for Research. Available at: https://eric.ed.gov/?id=ED613589

[ref006] Tsan J. Day S. (2007). Personality and gender as predictors of virtual counseling use.

[ref38] Worth A. K. (2022). The experiences of school counselors providing virtual services during Covid-19: A phenomenological investigation. Doctoral Dissertation. Counseling & Human Services, Old Dominion University.

[ref39] Zamani Z. A. Nasir R. Yusooff F. (2010). Perceptions towards virtual counseling among counselors in Malaysia. Procedia Soc. Behav. Sci. 5, 585–589. doi: 10.1016/j.sbspro.2010.07.146

